# The impact of SGLT2 inhibitors on uric acid levels in patients with heart failure with preserved ejection fraction: integration of mechanisms and clinical evidence

**DOI:** 10.3389/fcvm.2026.1726611

**Published:** 2026-08-07

**Authors:** Yuan Zhang, Xue Xiao, Qu Yan, Daping Huang, Wanqin Wang

**Affiliations:** 1 Department of Pharmacy, Zhejiang Provincial People's Hospital Bijie Hospital (The First People's Hospital of Bijie), Bijie, China; 2 Department of Emergency Medicine, Zhejiang Provincial People’s Hospital Bijie Hospital, Bijie, China; 3 Department of Cardiology, Zhejiang Provincial People's Hospital Bijie Hospital (The First People's Hospital of Bijie), Bijie, China

**Keywords:** bioinformatics, clinical data analysis, heart failure with preserved ejection fraction, hyperuricemia, machine learning, molecular docking, network pharmacology, SGLT2 inhibitors

## Abstract

**Objective:**

Hyperuricemia (HUA) is a prevalent comorbidity in patients with heart failure with preserved ejection fraction (HFpEF) and is closely associated with disease progression and adverse outcomes. Sodium-glucose cotransporter 2 (SGLT2) inhibitors can rapidly and sustainably reduce serum uric acid (SUA) levels and lower the incidence of HUA-related clinical events; however, their underlying mechanisms remain unclear. This study employed network pharmacology and molecular docking to systematically investigate the potential mechanisms of SGLT2 inhibitors in HFpEF patients with concomitant hyperuricemia, focusing on uric acid metabolism modulation and inflammation-related pathways. Additionally, a machine learning approach was applied to retrospectively analyze the effects of SGLT2 inhibitors on SUA levels, aiming to clarify the therapy’s biological basis and clinical relevance.

**Methods:**

The SMILES structures of eleven SGLT2 inhibitors were retrieved from PubChem. Potential drug targets were predicted via the SwissTargetPrediction platform and intersected with HFpEF- and HUA-related targets obtained from OMIM and GeneCards to identify common targets. Protein–protein interaction (PPI) networks were constructed using STRING to identify hub genes, followed by Gene Ontology (GO) and Kyoto Encyclopedia of Genes and Genomes (KEGG) enrichment analyses using DAVID. Molecular docking with AutoDock Vina assessed binding affinities. Clinical data of HFpEF patients were collected, and a classification model based on 34 clinical variables was developed using the FLAML automated machine learning framework to predict SGLT2 inhibitor treatment status and changes in SUA. Model interpretability and feature importance were performed using SHAP analysis.

**Results:**

Eleven SGLT2 inhibitors acted on 32 targets associated with SUA reduction in HFpEF patients. Key pathways included tumor necrosis factor (TNF), advanced glycation end product–receptor for advanced glycation end product (AGE–RAGE), interleukin-17 (IL-17), and Kaposi’s sarcoma-associated herpesvirus (KSHV) signaling. Molecular docking demonstrated favorable binding of SGLT2 inhibitors to core targets, including GAPDH, CASP3, ICAM1, ACE, DPP4, and AGTR1, with canagliflozin showing the strongest predicted binding affinity. Clinical data analyses identified uric acid and creatinine as the most important predictors in the machine learning model, reflecting their importance in model predictions.

**Conclusion:**

By integrating network pharmacology and retrospective clinical analysis, this study elucidates the potential uric acid–lowering mechanisms of SGLT2 inhibitors in HFpEF patients. The identified targets and pathways provide mechanistic insights into SGLT2 inhibitor pharmacology and support their therapeutic potential for managing hyperuricemia.

## 1 Introduction

Heart failure (HF) is the terminal stage of various cardiac diseases and has emerged as a major global public health burden (1, 2). Among its subtypes, heart failure with preserved ejection fraction (HFpEF) accounts for approximately 50% of all HF cases, with both incidence and prevalence continuing to rise worldwide (3). In recent years, hyperuricemia (HUA), a common metabolic disorder, has garnered increasing attention due to its detrimental effects on cardiovascular health (4–7). Substantial epidemiological evidence demonstrates that HUA is not only an independent risk factor for hypertension, coronary artery disease, and chronic kidney disease but is also closely associated with the onset and progression of HFpEF (8). More than 50% of HFpEF patients present with elevated baseline serum uric acid (SUA) levels (9–11), which correlate with disease severity and adverse prognosis. SUA, the final product of purine nucleotide metabolism catalyzed by xanthine oxidase, reflects both enzyme activity and the balance between purine intake and renal excretion. Elevated SUA contributes to endothelial dysfunction, impaired myocardial energetics, increased blood pressure, and reduced renal function, thereby exacerbating the progression of HF (12, 13). These pathophysiological processes further contribute to the development of HFpEF.

Previous studies have demonstrated that sodium–glucose cotransporter 2 (SGLT2) inhibitors reduce the risk of heart failure hospitalization and improve cardiovascular outcomes, significantly enhancing patients’ quality of life (12, 14). Large-scale randomized clinical trials have consistently demonstrated the cardiovascular benefits of individual SGLT2 inhibitors. Empagliflozin has been shown to reduce the risk of heart failure hospitalization and cardiovascular events across a broad range of ejection fraction phenotypes (15). Similarly, dapagliflozin has demonstrated significant reductions in worsening heart failure or cardiovascular death in patients with heart failure (16). In addition, ertugliflozin has shown potential cardioprotective effects, further supporting the class-wide benefit of SGLT2 inhibitors in heart failure management (17). Consequently, SGLT2 inhibitors have emerged as a promising therapeutic option for HFpEF (18, 19). Consequently, SGLT2 inhibitors have emerged as a promising therapeutic option for HFpEF (18, 19). Encouragingly, these agents have also been shown to decrease serum uric acid levels; however, their underlying mechanisms and clinical significance in HFpEF remain unclear (20). In this study, a network pharmacology approach was applied to construct a comprehensive drug–target–disease interaction network to systematically elucidate the molecular mechanisms of SGLT2 inhibitors and address the limitations of conventional single-target studies (21). Protein–protein interaction (PPI) networks were established to identify potential target proteins, followed by Gene Ontology (GO) functional annotation and Kyoto Encyclopedia of Genes and Genomes (KEGG) pathway enrichment analyses to explore relevant biological processes. Furthermore, a machine learning–based analysis of clinical data was conducted to evaluate the effect of SGLT2 inhibitors on SUA levels in patients with HFpEF, providing clinical validation for the network pharmacology results. This multidimensional and interdisciplinary strategy provides comprehensive theoretical and empirical evidence for the comorbidity management of HFpEF and HUA.

## 2 Materials and methods

### 2.1 Network pharmacology analysis

#### 2.1.1 Acquisition and integration of disease-associated targets

Potential disease-related targets associated with “heart failure with preserved ejection fraction (HFpEF)” and “hyperuricemia” were systematically retrieved from the OMIM (https://www.omim.org/) and GeneCards (https://www.genecards.org/) databases. The retrieved datasets were de-duplicated and integrated to construct a comprehensive repository of disease-associated targets for subsequent analyses.

#### 2.1.2 Prediction and screening of SGLT2 inhibitor targets

Eleven clinically approved SGLT2 inhibitors—canagliflozin, dapagliflozin, empagliflozin, luseogliflozin, tofogliflozin, ipragliflozin, ertugliflozin, enavogliflozin, henagliflozin, sotagliflozin, and bexagliflozin—were included for target prediction. Their SMILES structures were obtained from the PubChem database (https://pubchem.ncbi.nlm.nih.gov/) and imported into the SwissTargetPrediction platform (http://www.swisstargetprediction.ch/) to predict potential protein targets. Targets with a prediction probability greater than zero were retained. The intersection between drug-related and disease-associated targets was identified and visualized as Venn diagrams using the BioLadder online bioinformatics platform (https://www.bioladder.cn/web/), thereby identifying potential common targets for further analysis.

#### 2.1.3 Construction of the protein–protein interaction network and identification of core targets

The predicted common targets were uploaded to the STRING database (https://string-db.org/) with the organism restricted to *Homo sapiens* and a minimum interaction confidence score of 0.4. The interaction network was visualized using Cytoscape (version 3.10.0). Topological parameters, including degree, betweenness centrality, and closeness centrality, were analyzed using the CentiScaPe 2.2 plugin. Nodes exhibiting all three parameters above their mean values were defined as core targets and subsequently subjected to functional enrichment analysis.

Core targets were identified based on topological parameters, including degree, betweenness centrality, and closeness centrality, with thresholds defined as values exceeding the corresponding mean levels within the network. This approach is widely used in network pharmacology to prioritize relatively central nodes that may play key regulatory roles.

However, we acknowledge that the use of mean-based thresholds may be relatively permissive and could introduce potential noise. Therefore, the identified core targets should be interpreted as candidate targets rather than definitive functional regulators. Future studies incorporating stricter selection criteria or sensitivity analyses are warranted to further validate the robustness of these findings.

#### 2.1.4 Construction and topological analysis of the drug–target–disease network

A comprehensive “drug–target–disease” interaction network was constructed using Cytoscape (version 3.10.1). Distinct node types (drug, target, disease) were assigned unique attributes. Network parameters, including density, average clustering coefficient, and degree distribution, were calculated to evaluate the network’s structural properties and stability, thereby elucidating the mechanisms underlying drug–target–disease interactions.

#### 2.1.5 Functional enrichment analysis

The identified core targets were submitted to the DAVID database (https://davidbioinformatics.nih.gov/) for Gene Ontology (GO) annotation and Kyoto Encyclopedia of Genes and Genomes (KEGG) pathway enrichment analyses. GO terms and pathways with both p-values and q-values < 0.05 were considered statistically significant. The top 10 enriched GO terms and top 20 KEGG pathways were visualized using the Bioinformatics online platform (https://www.bioinformatics.com.cn/) to highlight the major biological processes and signaling pathways involved.

#### 2.1.6 Molecular docking validation

Molecular docking was performed between the active SGLT2 inhibitor compounds and the core target proteins identified from the network analysis. Three-dimensional structures of the ligands were obtained from the PubChem database (https://pubchem.ncbi.nlm.nih.gov/), and protein crystal structures were downloaded from the Protein Data Bank (PDB, https://www.rcsb.org/). Protein preprocessing was conducted using AutoDockTools (version 1.5.7) by removing water molecules, adding polar hydrogens, and assigning Gasteiger charges. Docking simulations were executed in AutoDock Vina, with the grid box centered on the active binding pocket. The lowest binding energy of each ligand–receptor complex was recorded. PyMOL (version 2.4) was used for three-dimensional visualization of binding conformations, hydrogen-bond interactions, key amino acid residues, and bond lengths.

### 2.2 Clinical data analysis based on machine learning

#### 2.2.1 Inclusion criteria

Patients diagnosed with heart failure with preserved ejection fraction (HFpEF) who were hospitalized in the Department of Cardiology, Zhejiang Provincial People’s Hospital Bijie Hospital, between November 2021 and December 2022, were included in this study. The inclusion and exclusion criteria were formulated in accordance with relevant clinical guidelines and research objectives, encompassing age range, diagnostic standards, comorbidities, and prior treatment history, to ensure both representativeness and data completeness. This study was approved by the Ethics Committee of Zhejiang Provincial People’s Hospital Bijie Hospital (Approval No. [2023-12-24(2)]) and conducted in compliance with the Declaration of Helsinki. Ethical approval was not additionally required because only secondary, de-identified, and publicly available data were used.

**Inclusion Criteria:**

1. Age ≥ 18 years;
2. Meeting the diagnostic criteria for HFpEF according to the
3. Meeting the diagnostic criteria for **heart failure with preserved ejection fraction (HFpEF)** according to the *Chinese Guidelines for the Diagnosis and Treatment of Heart Failure (2018)*, with hemodynamic stability;
4. Classified as **New York Heart Association (NYHA)** class II–IV;
5. Treatment duration of 6–24 months;
6. Underwent at least two of the following assessments before and after treatment—echocardiography, electrocardiography, NT-proBNP, serum biomarkers, Minnesota Living with Heart Failure Questionnaire (MLHFQ), or six-minute walk test—with an interval of at least six months between evaluations.

**Exclusion Criteria:**

1. Blood pressure <90/60 mmHg;
2. Moderate to severe renal impairment (eGFR <60 mL/min/1.73 m${}^{2}$);
3. Life expectancy <1 year or presence of advanced malignancy;
4. Severe malnutrition.

#### 2.2.2 Data collection

Comprehensive multidimensional clinical data were collected, including 34 variables covering hematological, biochemical, inflammatory, echocardiographic, and vital-sign parameters. Specific indicators comprised hemoglobin, platelet count, uric acid, creatinine, urea, glucose, triglycerides, high- and low-density lipoprotein cholesterol, glycated hemoglobin, troponin, myoglobin, creatine kinase-MB, C-reactive protein, high-sensitivity C-reactive protein, interleukin-6, left ventricular diastolic function, right ventricular outflow tract diameter, left ventricular end-diastolic and end-systolic diameters, right atrial and ventricular diameters, left atrial diameter, left ventricular ejection fraction, interventricular septal and posterior wall thickness, as well as heart rate, systolic, and diastolic blood pressure at both admission and discharge.

#### 2.2.3 Data organization and preprocessing

A total of 398 patients were enrolled and categorized into treatment and control groups according to SGLT2 inhibitor therapy status, enabling comprehensive evaluation of therapeutic efficacy. Among them, 34 patients with complete pre- and post-treatment records were selected, yielding 68 paired samples for individual-level effect analysis. Missing data were handled using a complete-case analysis strategy. For continuous variables, records with missing values were excluded from statistical analyses. Categorical variables with missing values were excluded from corresponding analyses. Categorical variables were converted to numerical features via one-hot encoding, and continuous variables were standardized to eliminate dimensional disparities and ensure data consistency.

#### 2.2.4 Model construction and evaluation

Automated machine learning (AutoML) was implemented using FLAML 2.3.4 (https://microsoft.github.io/FLAML/) (22) to develop classification models. Five-fold stratified cross-validation was employed to maintain class balance between training and validation sets, thereby enhancing model robustness and generalizability. During preprocessing, categorical variables were converted to categorical data types and continuous variables to floating-point types to improve compatibility and computational efficiency. FLAML automatically searched for the optimal learning algorithm and hyperparameter configuration using the area under the receiver operating characteristic curve (ROC AUC) on the validation set as the optimization metric. Each fold was trained under a fixed time budget with a unified random seed to ensure reproducibility. Two independent exploratory classification tasks were conducted: (1) identifying patterns associated with SGLT2 inhibitor treatment status, and (2) distinguishing pre- and post-treatment samples for hypothesis-generating analysis rather than confirmatory treatment effect estimation. Model performance was evaluated based on the mean ROC AUC across five folds.

#### 2.2.5 Model interpretation and target analysis

The SHAP (SHapley Additive exPlanations) method (23), grounded in cooperative game theory, was employed to quantify the contribution of each input feature to model predictions, thereby elucidating the underlying decision-making mechanisms. This interpretability analysis identified key predictive features exerting substantial influence, offering insights into potential biological markers or therapeutic targets and supporting subsequent mechanistic and clinical strategy development.

## 3 Results

### 3.1 Network pharmacology analysis results

#### 3.1.1 Disease-related target screening

**Figure 1:**
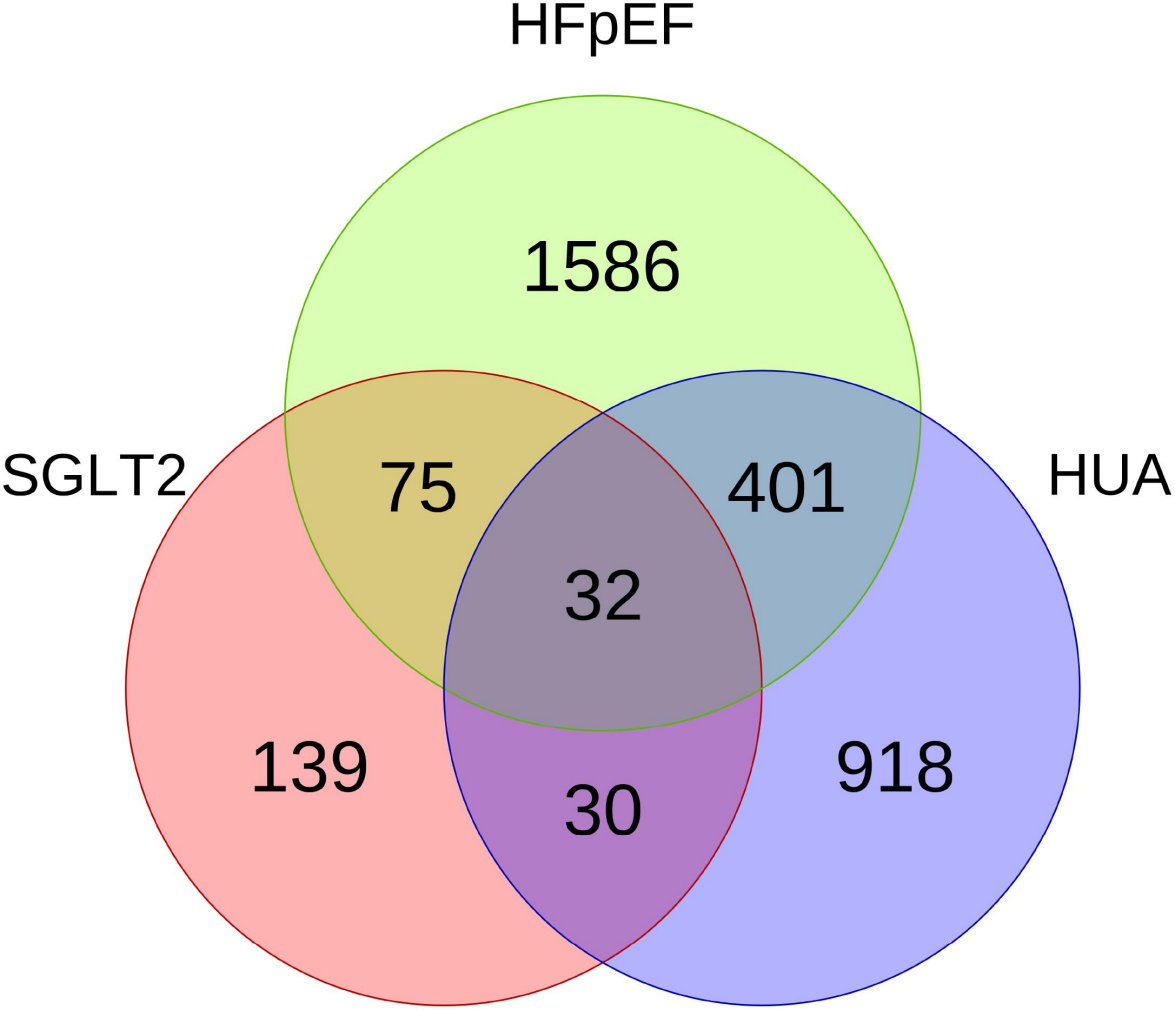
Venn diagram showing overlapping targets of SGLT2 inhibitors, HFpEF, and hyperuricemia. Red nodes represent SGLT2 inhibitor targets, green nodes represent HFpEF targets, and blue nodes represent HUA targets.

A total of 2,095 targets associated with HFpEF and 1,381 targets associated with hyperuricemia (HUA) were retrieved from the OMIM and GeneCards databases. After further filtering, 32 overlapping targets associated with SGLT2 inhibitors, HFpEF, and HUA were identified (Figure 1). The overlap was visualized using a Venn diagram, with distinct colors representing the target sets corresponding to SGLT2 inhibitors, HFpEF, and HUA.

#### 3.1.2 Protein–protein interaction (PPI) network analysis

**Figure 2:**
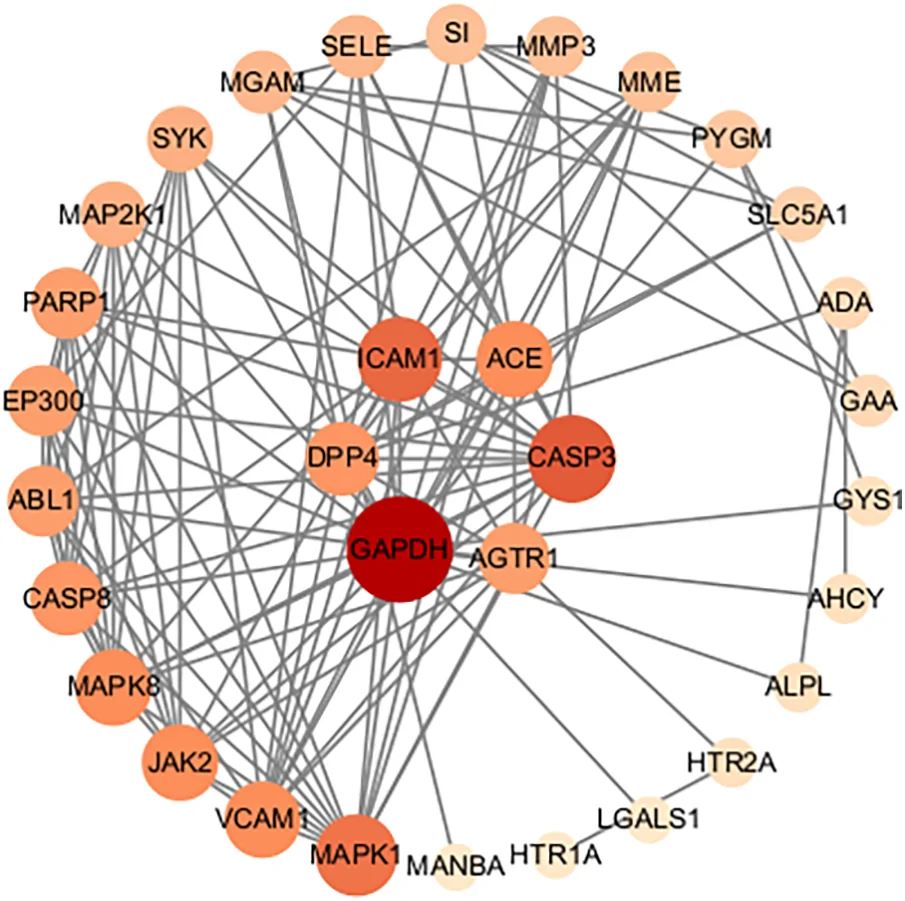
PPI network. Node size and color represent degree, and edge width reflects interaction strength.

The 32 overlapping targets were imported into the STRING database to obtain protein–protein interaction information and construct a PPI network. In the visualization, node size and color intensity correspond to node degree, while edge thickness reflects interaction confidence (Figure 2). Key hub targets, including GAPDH, CASP3, ICAM1, ACE, DPP4, and AGTR1, were identified, with detailed degree values provided in Table 1.

**Table 1:** Network topology parameters of core targets.

Target	Betweenness	Closeness	Degree
ABL proto-oncogene 1 (ABL1)	6.7048	0.0161	10
Mitogen-activated protein kinase 1 (MAPK1)	26.7519	0.0192	14
E1A binding protein p300 (EP300)	3.8603	0.0161	10
Vascular cell adhesion molecule 1 (VCAM1)	22.2839	0.0189	12
Caspase 3 (CASP3)	44.0117	0.0196	16
Caspase 8 (CASP8)	2.5349	0.0164	11
Poly(ADP-ribose) polymerase 1 (PARP1)	1.2659	0.0161	10
Mitogen-activated protein kinase 8 (MAPK8)	11.0849	0.0172	12
Glyceraldehyde-3-phosphate dehydrogenase (GAPDH)	345.0405	0.0244	23
Angiotensin II receptor type 1 (AGTR1)	123.9938	0.0185	10
Adenosylhomocysteinase (AHCY)	6.3548	0.0145	2
Janus kinase 2 (JAK2)	10.8571	0.0172	12
Matrix metallopeptidase 3 (MMP3)	0.2857	0.0152	6
Glucosidase, alpha; acid (GAA)	0.0000	0.0119	3
Galectin 1 (LGALS1)	0.0000	0.0141	1
5-hydroxytryptamine receptor 1A (HTR1A)	0.0000	0.0089	1
Membrane metalloendopeptidase (MME)	4.3000	0.0169	6
Spleen tyrosine kinase (SYK)	0.9802	0.0156	8
Angiotensin-converting enzyme (ACE)	106.4049	0.0196	12
Sucrase-isomaltase (SI)	14.1667	0.0141	6
Intercellular adhesion molecule 1 (ICAM1)	39.4371	0.0200	15
Sodium/glucose cotransporter 1 (SLC5A1)	0.0000	0.0135	4
Maltase-glucoamylase (MGAM)	74.1667	0.0143	7
E-selectin (SELE)	3.8526	0.0164	7
Dipeptidyl peptidase 4 (DPP4)	121.5711	0.0192	11
Adenosine deaminase (ADA)	7.3333	0.0128	3
Alkaline phosphatase (ALPL)	6.3548	0.0145	2
Mitogen-activated protein kinase kinase 1 (MAP2K1)	0.0000	0.0156	8
Glycogen phosphorylase, muscle associated (PYGM)	52.4026	0.0164	5
Glycogen synthase 1 (GYS1)	0.0000	0.0152	2
Beta-mannosidase (MANBA)	0.0000	0.0100	1
5-hydroxytryptamine receptor 2A (HTR2A)	60.0000	0.0122	2

**Figure 3:**
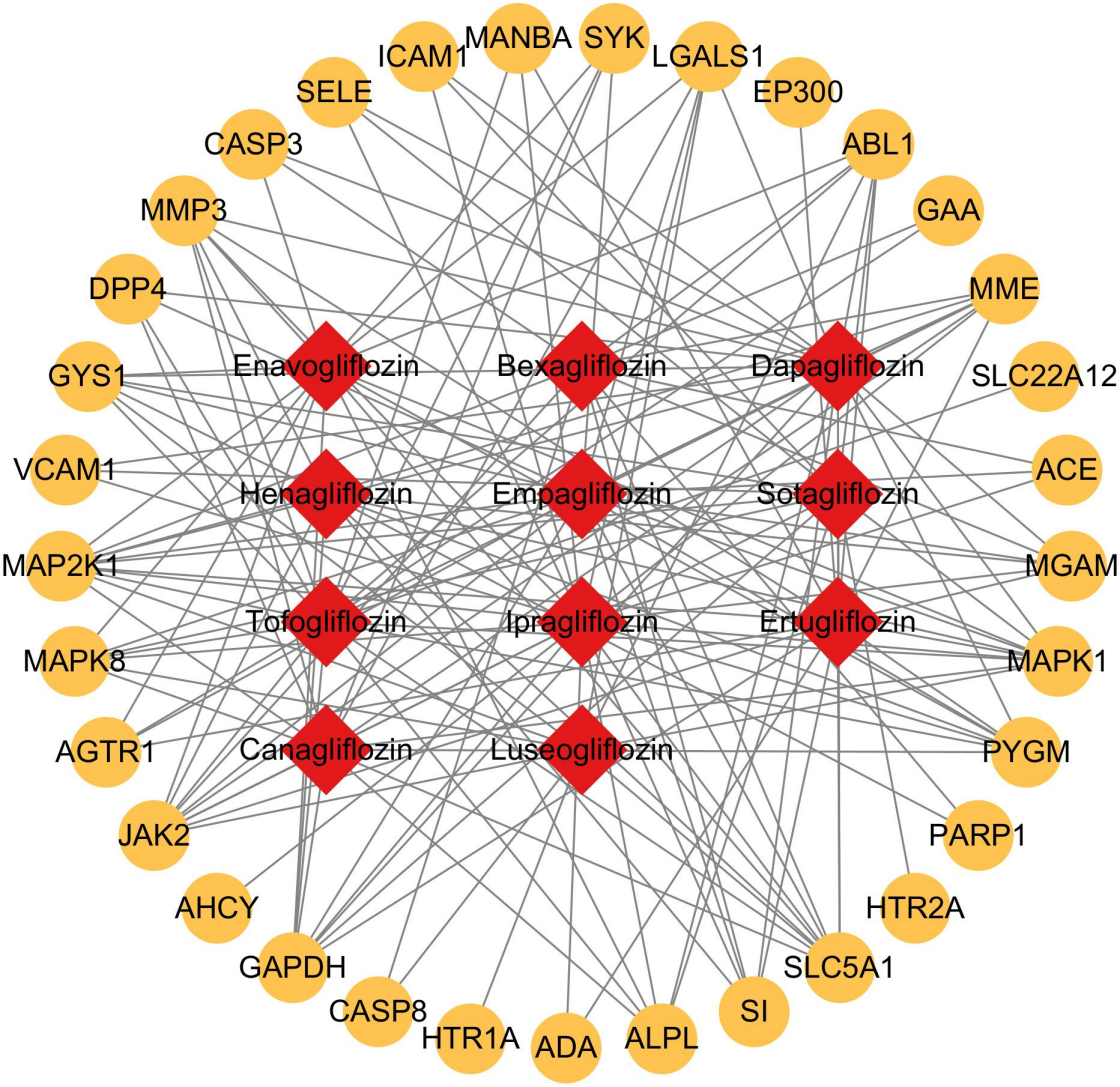
Drug–target–disease network. Red nodes represent SGLT2 inhibitors, and yellow nodes represent disease-related targets.

A drug–target–disease regulatory network was subsequently constructed using Cytoscape (v3.10.1), comprising 44 nodes (32 overlapping targets and 11 SGLT2 inhibitors) and 163 interactions. In Figure 3, red nodes represent SGLT2 inhibitors, and yellow nodes indicate disease-related targets. These results suggest that SGLT2 inhibitors may exert a multi-target synergistic effect in the treatment of HFpEF concomitant with HUA.

#### 3.1.3 Functional enrichment analysis

**Figure 4:**
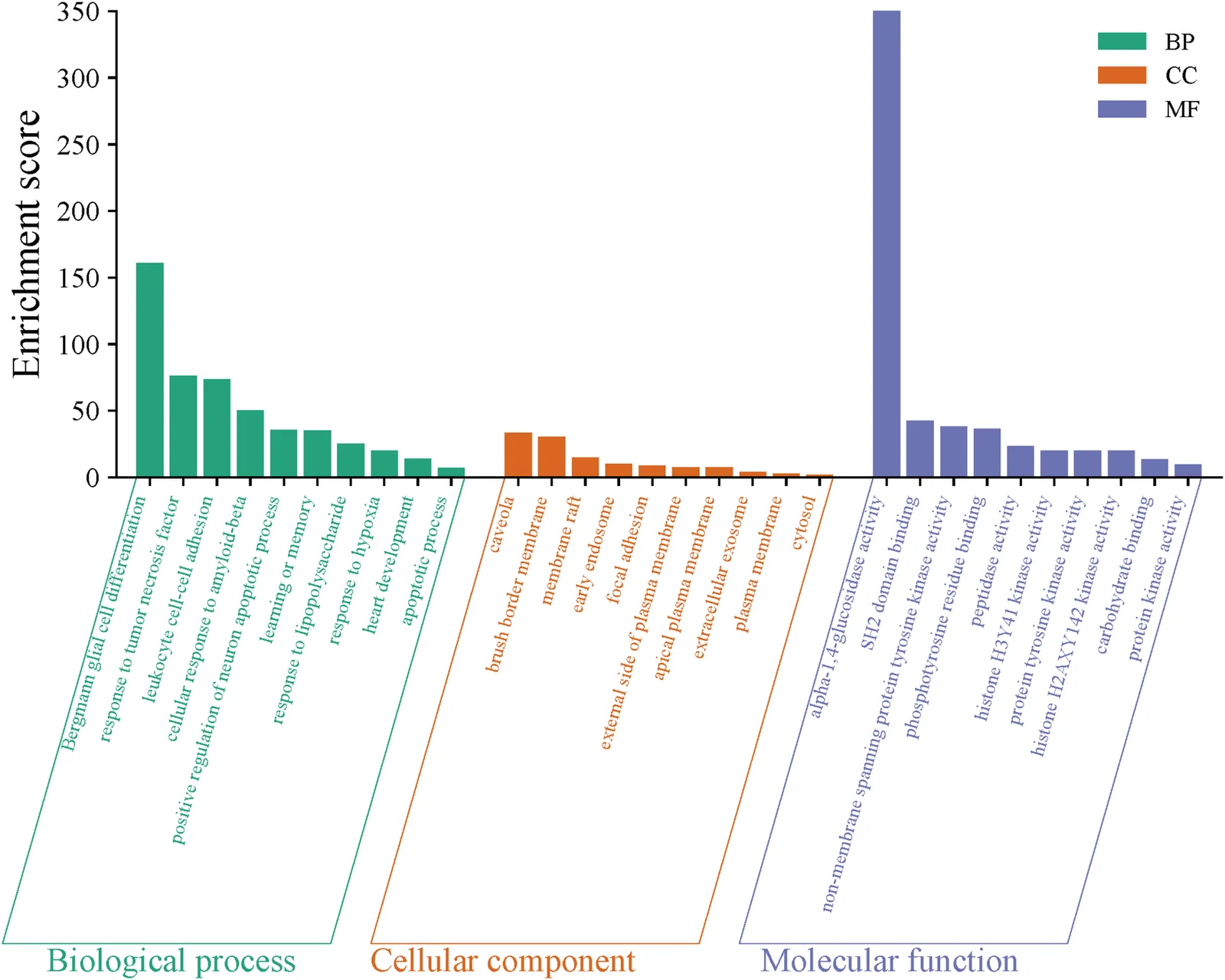
GO functional enrichment analysis. The bar chart displays major enriched biological processes, cellular components, and molecular functions.

**Figure 5:**
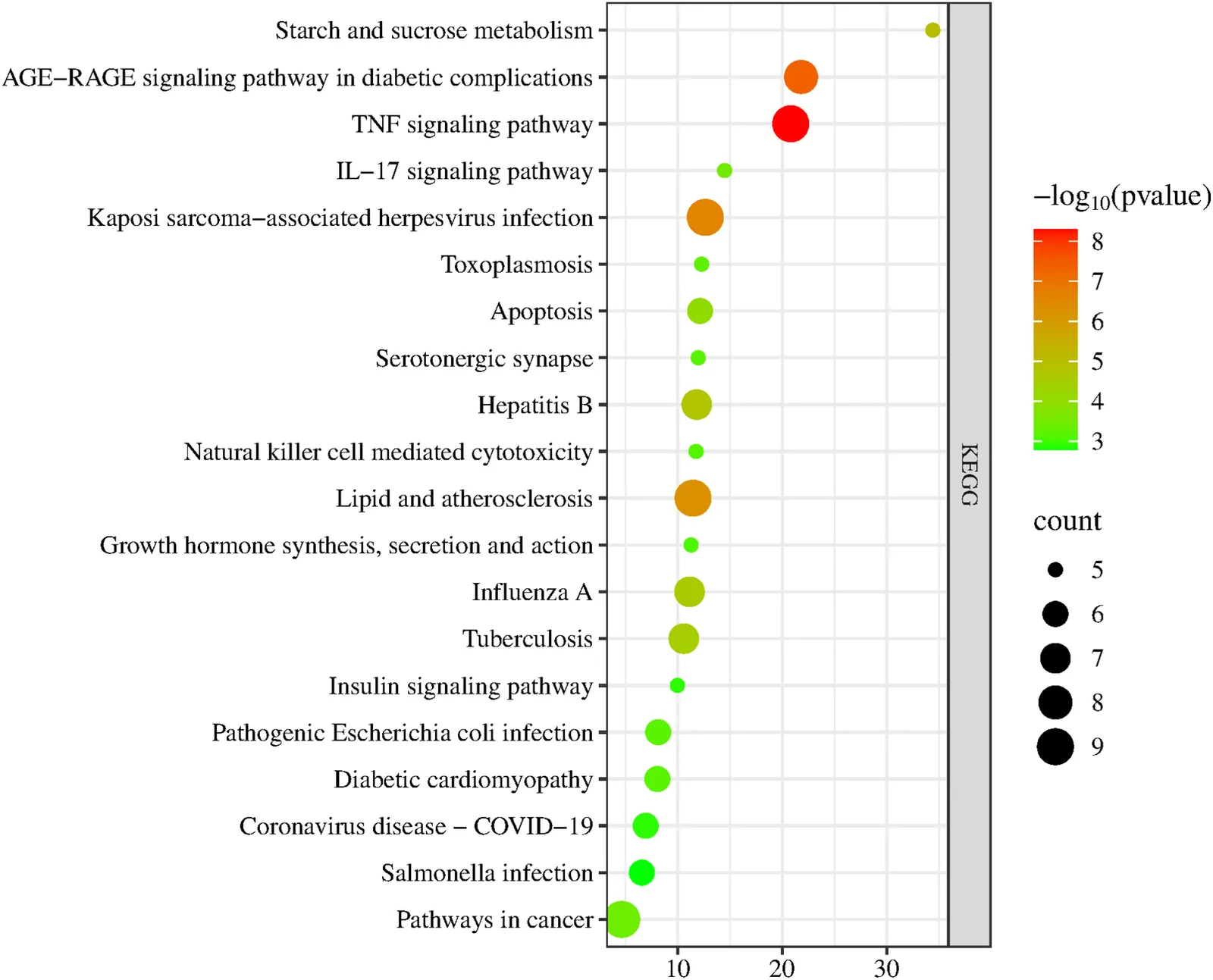
KEGG pathway enrichment analysis. Color intensity indicates the degree of target enrichment in each pathway.

GO annotation was performed for biological process (BP), cellular component (CC), and molecular function (MF) categories. Significantly enriched terms included responses to lipopolysaccharide, regulation of apoptotic processes, membrane regions, and various enzyme activities (Figure 4). KEGG pathway enrichment analysis revealed that core targets were significantly enriched in signaling pathways closely associated with cardiovascular disease and metabolic dysregulation, including the TNF, AGE–RAGE, and IL-17 pathways (Figure 5).

#### 3.1.4 Molecular docking analysis

Representative active components of the 11 SGLT2 inhibitors were docked with hub targets (GAPDH, CASP3, ICAM1, ACE, DPP4, and AGTR1) using AutoDock Vina. Lower binding energies indicate greater stability of the ligand–receptor complex; binding energies below −5.0 kcal/mol indicate favorable binding, whereas energies below −7.0 kcal/mol indicate strong affinity.

All active components exhibited binding energies below −5 kcal/mol, suggesting favorable predicted interactions with the core targets in silico (Table 2). Representative docking conformations indicated that hydrogen bonding and hydrophobic interactions may contribute to ligand–target stabilization. These results represent computational predictions rather than experimentally validated molecular interactions and should be interpreted as hypothesis-generating.

**Table 2:** Binding energies of major active components of SGLT2 inhibitors with core targets (kcal/mol).

Drug	GAPDH	CASP3	ICAM1	ACE	DPP4	AGTR1
Canagliflozin	−9.1	−8.8	−6.2	−10.7	−9.3	−10.0
Dapagliflozin	−7.7	−7.7	−8.0	−9.4	−8.1	−8.0
Ipragliflozin	−8.6	−7.6	−6.6	−9.5	−8.7	−9.0
Ertugliflozin	−8.4	−7.7	−7.5	−10.0	−8.7	−8.4
Enavogliflozin	−7.6	−8.3	−7.2	−10.3	−8.9	−8.8
Luseogliflozin	−7.5	−7.3	−7.1	−8.2	−7.8	−8.0
Henagliflozin	−7.5	−7.7	−7.1	−9.6	−8.9	−8.2
Tofogliflozin	−7.8	−8.0	−7.1	−10.4	−8.4	−8.9
Sotagliflozin	−7.7	−7.2	−6.5	−7.4	−8.1	−8.4
Empagliflozin	−8.0	−7.9	−7.0	−9.9	−8.5	−8.4
Bexagliflozin	−6.7	−7.7	−8.0	−9.1	−8.1	−8.3

**Figure 6:**
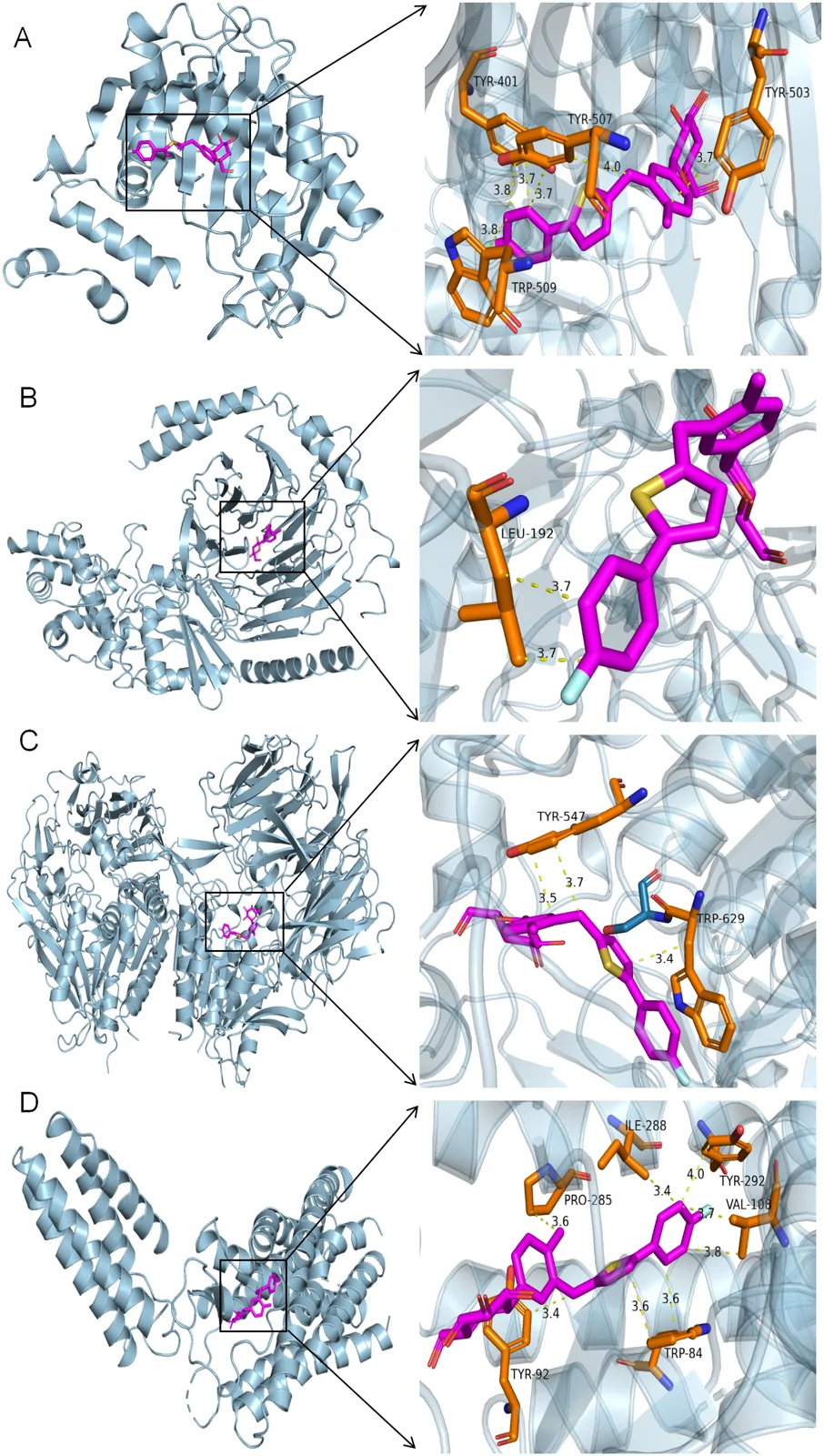
Molecular docking visualization of representative active components with core targets, showing binding modes and key hydrogen bond interactions. Panel **A**: GAPDH–Canagliflozin; Panel **B**: ACE–Canagliflozin; Panel **C**: DPP4–Canagliflozin; Panel **D**: AGTR1–Canagliflozin. Dashed lines indicate hydrogen bonds, and values represent bond lengths. Residue names indicate binding sites.

Representative low-energy conformations were visualized using PyMOL to illustrate key hydrogen bond interactions and binding residues (Figure 6).

Further experimental validation, such as biochemical binding assays or functional studies, is required to confirm the biological relevance of these predicted interactions.

### 3.2 Results of machine learning model analysis

#### 3.2.1 Clinical data statistical analysis

Multiple clinical indicators were compared between the SGLT2 inhibitor treatment group and the control group. Several variables exhibited statistically significant differences between the two groups (p<0.05). Specifically, hemoglobin levels were significantly higher in the treatment group compared with the control group (139.19 vs. 130.78 g/L, p=0.0003), urea levels were lower (6.39 vs. 7.68 mmol/L, p=0.0004), left ventricular ejection fraction increased (59.14% vs. 57.00%, p=0.0086), and creatinine levels decreased (81.00 vs. 135.81 μμmol/L, p=0.0091). Additionally, creatine kinase isoenzyme (16.69 vs. 15.24 U/L, p=0.0181), C-reactive protein (18.34 vs. 32.33 mg/L, p=0.0245), and blood glucose (11.43 vs. 10.24 mmol/L, p=0.0387) also differed significantly. Most other indicators, including right atrial diameter, troponin, platelet count, and interleukin-6, showed no significant differences (p>0.05). These results provide key feature variables for subsequent predictive model development (Table 3).

**Table 3:** Comparison of key clinical indicators between treatment and control groups and pre- and post-intervention within the treatment group.

Variable	Control	Treatment	Between-group p	Pre-intervention	Post-intervention	Paired p
Hemoglobin (g/L)	130.78	139.19	0.0003	138.94	138.97	0.9957
Urea (mmol/L)	7.68	6.39	0.0004	7.11	7.45	0.6468
Left Ventricular EF (%)	57.00	59.14	0.0086	58.44	56.32	0.1769
Creatinine (μμmol/L )	135.81	81.00	0.0091	90.94	98.91	0.4781
Creatine Kinase Isoenzyme (U/L)	15.24	16.69	0.0181	18.60	12.18	0.2114
C-reactive Protein (mg/L)	32.33	18.34	0.0245	4.48	36.61	0.0703
Glucose (mmol/L)	10.24	11.43	0.0387	12.19	9.91	0.1710
Right Atrial Diameter (mm)	44.43	42.10	0.0936	43.67	42.10	0.4492
Left Ventricular Diastolic Function	1.00	0.91	0.1412	–	–	–
Troponin (ng/mL)	0.25	0.48	0.2563	0.0193	0.0397	0.1873
Right Ventricular Diameter (mm)	20.39	20.72	0.2658	20.65	20.76	0.8701
Left Atrial Diameter (mm)	33.37	34.14	0.2664	35.03	35.29	0.8765
Right Ventricular Outflow Tract Diameter (mm)	28.18	27.69	0.2750	27.15	29.09	0.0244
Left Ventricular End-Systolic Diameter (mm)	31.33	30.95	0.3939	31.56	31.15	0.6280
Myoglobin (ng/mL)	86.04	91.30	0.4106	65.18	107.97	0.3659
HDL-C (mmol/L)	1.17	1.15	0.4265	1.22	1.17	0.3791
HbA1c (%)	7.79	7.94	0.4313	7.87	8.02	0.7568
Admission Heart Rate (bpm)	78.88	80.13	0.4705	79.63	80.27	0.8612
LDL-C (mmol/L)	2.82	2.88	0.5455	2.70	2.59	0.6408
Uric Acid (μμmol/L)	369.89	377.64	0.5504	391.53	356.62	0.2532
Triglycerides (mmol/L)	2.33	2.45	0.5535	1.94	1.85	0.7672
Discharge Heart Rate (bpm)	74.72	74.14	0.5672	74.57	75.63	0.6561
Interventricular Septum Thickness (mm)	9.34	9.26	0.5786	9.29	8.71	0.0625
Admission Diastolic BP (mmHg)	81.69	80.88	0.6019	80.90	79.33	0.6582
Left Ventricular Posterior Wall Thickness (mm)	8.75	8.69	0.6511	8.53	8.29	0.4026
Interleukin-6 (pg/mL)	31.88	27.88	0.7280	38.08	51.70	0.7903
Discharge Diastolic BP (mmHg)	75.90	75.63	0.7587	74.57	76.53	0.3735
Platelet Count ($10^{9}$/L)	214.88	212.99	0.7985	207.44	196.09	0.4716
Admission Systolic BP (mmHg)	137.57	137.13	0.8537	141.17	136.63	0.4850
Discharge Systolic BP (mmHg)	125.56	125.65	0.9462	127.67	125.97	0.5895
Left Ventricular End-Diastolic Diameter (mm)	44.99	44.99	0.9934	45.44	44.09	0.1486

Within the SGLT2 inhibitor treatment group, paired-sample t-tests were performed for 34 patients comparing pre- and post-intervention clinical indicators. A significant increase was observed in right ventricular outflow tract diameter (29.09 mm vs. 27.15 mm, p=0.0244), whereas other indicators did not change significantly. Although creatinine (98.91 vs. 90.94 μμmol/L, p=0.4781) and uric acid (356.62 vs. 391.53 μμmol/L, p=0.2532) fluctuated, these changes were not statistically significant. Hemoglobin levels remained stable (138.97 vs. 138.94 g/L, p=0.9957). These findings suggest that short-term intervention may benefit certain cardiac structural parameters but has limited impact on overall biochemical indicators. Detailed statistical results are provided in Table 3.

#### 3.2.2 Machine learning model construction and performance evaluation

**Figure 7:**
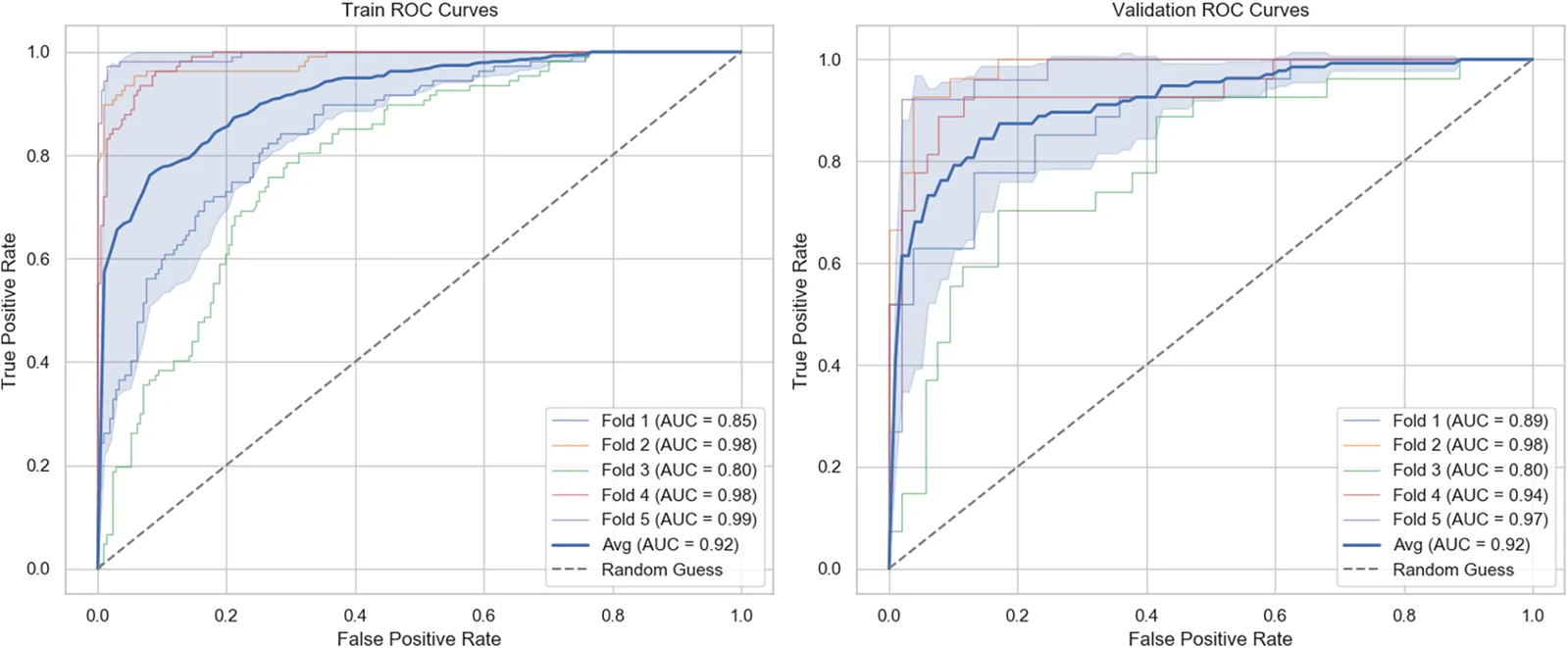
ROC curves of the model predicting SGLT2 inhibitor treatment status (treatment vs. control).

**Figure 8:**
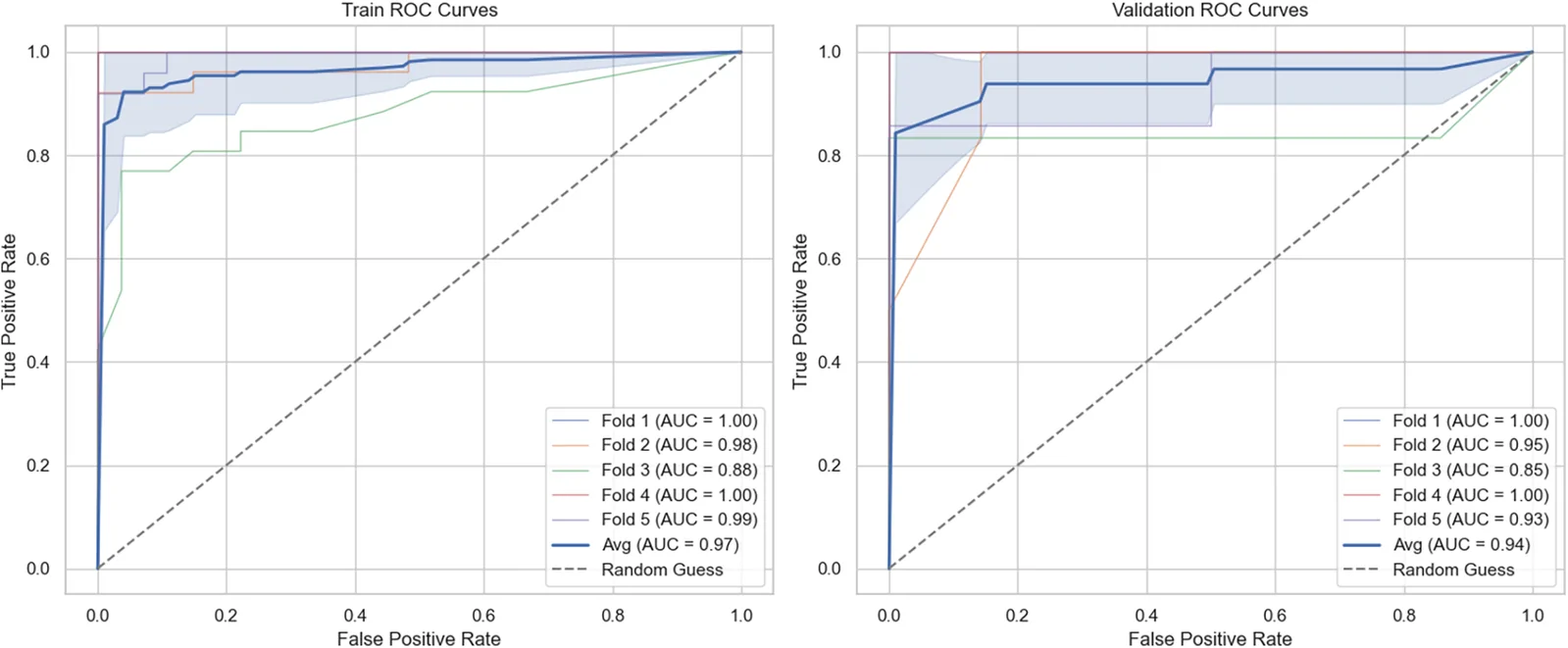
ROC curves of the model predicting changes in serum uric acid levels before and after treatment.

Using the 34 clinical features described above, the FLAML automated machine learning framework was employed to develop multiple classification models to explore associations between multidimensional clinical features, SGLT2 inhibitor treatment status, and serum uric acid variation. These analyses were conducted for hypothesis generation rather than causal inference. Through five-fold cross-validation, the model predicting treatment status achieved an average AUC of 0.92 on the validation set, indicating good discriminatory performance within this retrospective dataset, which should be interpreted cautiously given the limited sample size. (Figure 7). The model predicting changes in serum uric acid levels demonstrated even better performance, with an average AUC of 0.94 (Figure 8), indicating strong discriminative capability.

#### 3.2.3 SHAP-based interpretability analysis

**Figure 9:**
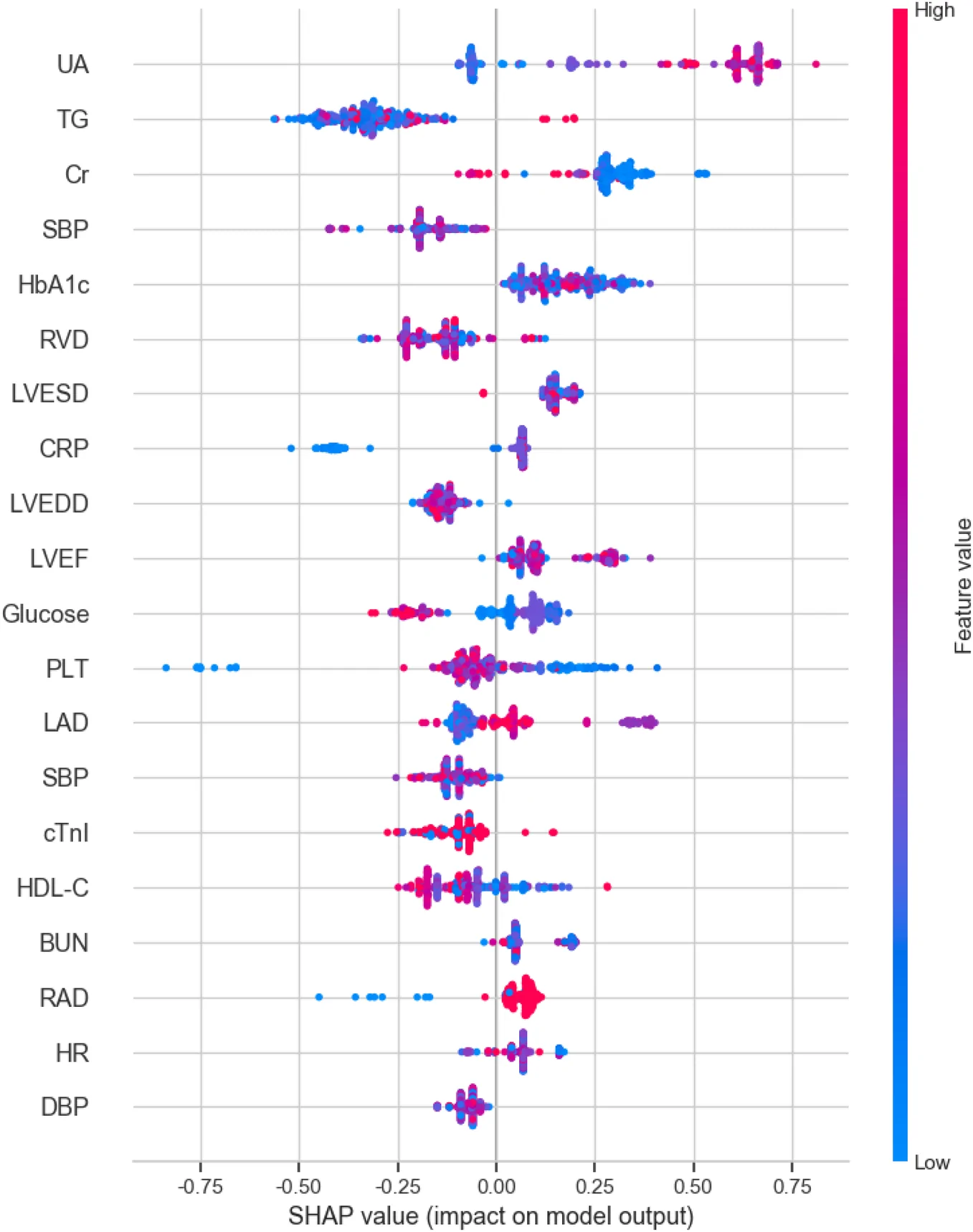
SHAP feature importance for predicting SGLT2 inhibitor treatment (treatment vs. control).

To enhance clinical interpretability, SHAP (SHapley Additive exPlanations) was applied to quantify the contribution of each clinical feature to model predictions (23). As shown in Figure 9, serum creatinine and uric acid were the most important variables for predicting SGLT2 inhibitor treatment. Higher creatinine levels corresponded to elevated SHAP values, indicating that the model was more likely to classify patients with impaired renal function as treated, consistent with the renal protective indication of SGLT2 inhibitors. Elevated uric acid levels also increased the predicted probability of treatment, suggesting that these variables were strongly associated with treatment allocation patterns in this cohort, without implying a causal treatment effect.

**Figure 10:**
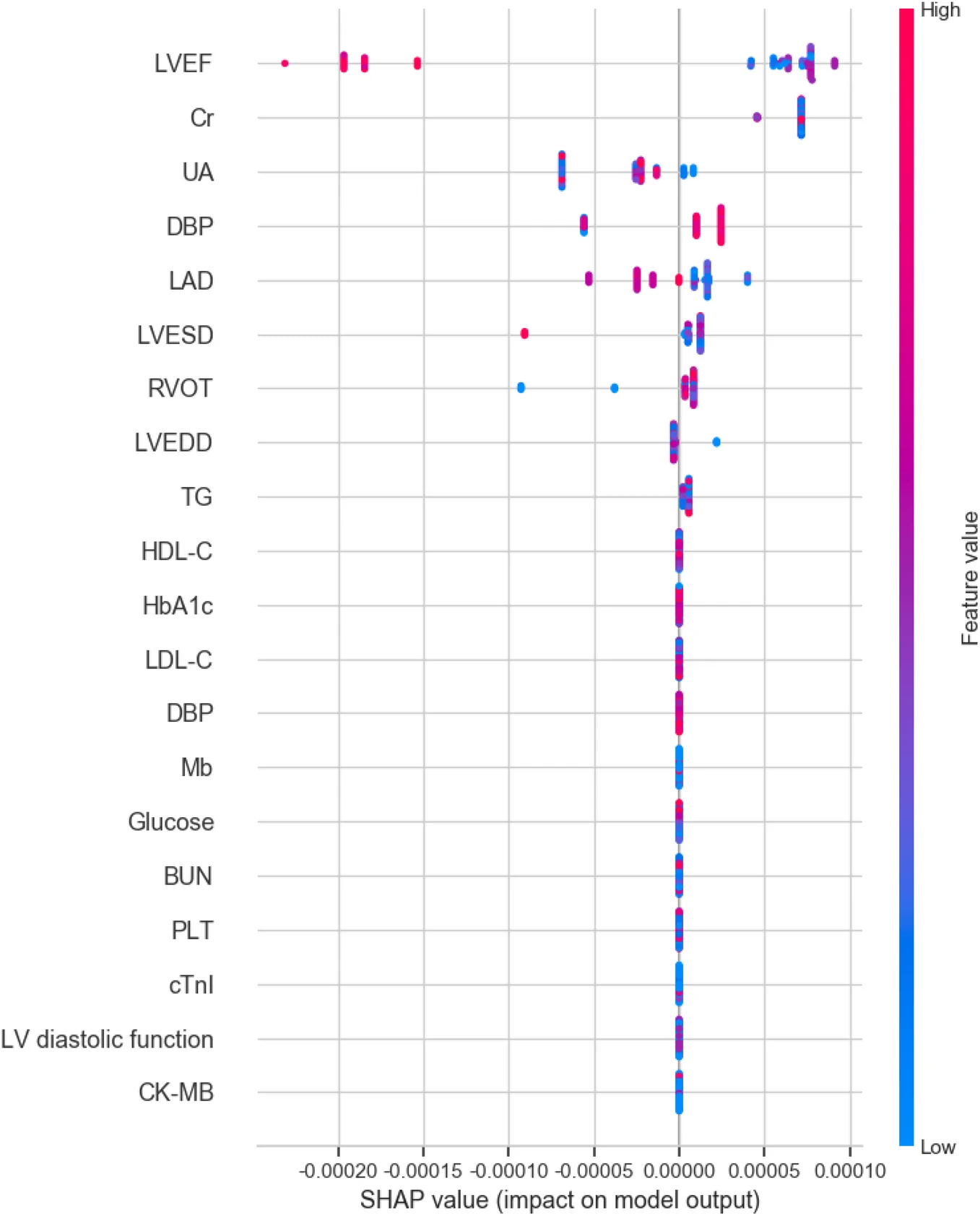
SHAP feature importance for predicting changes in serum uric acid levels before and after treatment.

For the model predicting changes in serum uric acid, Figure 10 highlights right ventricular outflow tract diameter, uric acid, and interleukin-6 as key contributing factors. Uric acid exhibited a strong positive SHAP contribution, highlighting its importance within the model’s predictive structure. Creatinine also contributed positively, indicating that the models effectively captured renal function-related changes. Additionally, inflammatory marker interleukin-6, metabolic indicator glucose, and cardiac structural parameter left ventricular end-diastolic diameter influenced model predictions, demonstrating the model’s capacity to integrate multi-dimensional clinical information, including inflammation, metabolism, and cardiac structure.

## 4 Discussion

### 4.1 HFpEF and HUA comorbidity mechanisms

Heart failure with preserved ejection fraction (HFpEF) and hyperuricemia (HUA) are interrelated conditions that mutually influence prognosis. Our network pharmacology analysis identified 1,381 common targets associated with HFpEF and HUA, enriched in oxidative stress, apoptosis, and inflammation-related pathways, providing a molecular basis for the comorbidity.

GAPDH participates in glycolysis and also functions as a cellular sensor, mediating mitochondrial signaling and apoptosis (24–27). Caspase-3, as a key executioner of apoptosis, mediates cardiomyocyte death and contributes to cardiac dysfunction (20, 28). ICAM1 is involved in inflammatory responses and immune regulation, linking HFpEF and HUA pathophysiology (29, 30). ACE, a key enzyme of RAAS, may promote myocardial remodeling and vascular stiffness in HFpEF (31). DPP4 modulates inflammation and fibrosis via cleavage of multiple substrates, indirectly affecting cardiovascular health (32–34). AGTR1 influences cardiac immune microenvironment and participates in HFpEF development (35).

Approximately 49% of HFpEF patients have hyperuricemia (female SUA > 5.7 mg/dL, male SUA > 7.0 mg/dL). Elevated serum uric acid is associated with advanced HF severity. These data suggest that HUA contributes to HFpEF risk and progression.

### 4.2 Mechanistic insights of SGLT2 inhibitors

SGLT2 inhibitors may modulate uric acid metabolism and mitigate inflammation induced by urate crystals (12). Molecular docking demonstrated high binding affinities of 11 SGLT2 inhibitors to the core targets (GAPDH, CASP3, ICAM1, ACE, DPP4, AGTR1), with canagliflozin potentially being the most effective component (36, 37).

In addition to GAPDH-mediated anti-inflammatory effects, SGLT2 inhibitors have been reported to decrease ICAM-1 expression in both animal models and patients with diabetes. This reduction likely mitigates endothelial inflammation, protects the vascular system, and contributes to the cardiovascular benefits observed in HFpEF patients. Thus, modulation of ICAM-1 may represent an additional mechanism by which SGLT2 inhibitors exert multi-targeted protective effects.

Clinical studies show that SGLT2 inhibitors reduce serum uric acid in T2DM patients, e.g., empagliflozin decreased SUA by 46 μmol/L (37), dapagliflozin by 39.798 μmol/L (38), ipragliflozin by 11.89-52.3 μmol/L (39), canagliflozin by 23.3 μmol/L (40), and tofogliflozin by 17.226 ± 6.237 μmol/L (41).

### 4.3 Clinical findings

In our retrospective cohort, SGLT2 inhibitor-treated patients showed improvements in hemoglobin, urea, left ventricular ejection fraction, and serum creatinine compared with controls. Prior meta-analyses of randomized trials have demonstrated that SGLT2 inhibitors improve composite outcomes such as heart failure hospitalization or cardiovascular death in patients with HFpEF, though effects on specific biomarkers vary across studies (42, 43). However, no statistically significant reduction in serum uric acid was observed in our cohort. This finding may differ from prior reports and could be attributable to the limited sample size, retrospective design, and population heterogeneity. Therefore, our clinical findings should be interpreted as exploratory and hypothesis-generating rather than confirmatory. Paired-sample analysis suggested potential right heart remodeling, reflected by an increase in right ventricular outflow tract diameter, supporting the need for longitudinal evaluation.

### 4.4 Machine learning-based analysis

Using the FLAML automated machine learning framework, predictive models achieved high accuracy for medication status and SUA variation (AUC = 0.85 and 0.92). SHAP analysis identified creatinine and uric acid as the most influential predictors, highlighting their importance in SGLT2 inhibitor pharmacodynamics. Additional predictors included interleukin-6 and cardiac structural indices (e.g., RVOT diameter, LVEDD), suggesting multi-pathway benefits through anti-inflammatory effects and cardiac remodeling (44).

### 4.5 Limitations and future directions

Limitations of this study include a relatively small sample size, particularly for paired pre- and post-intervention data, and a short observation period precluding assessment of long-term efficacy and cardiac remodeling. Additionally, due to the limited paired sample size and retrospective design, multivariable adjusted modeling could be statistically unstable and prone to overfitting. Therefore, the clinical findings are presented as descriptive rather than inferential. Future studies should involve multicenter, large-scale trials with long-term follow-up, complemented by functional validation (e.g., gene knockdown, overexpression, or pathway inhibition) to further elucidate the molecular mechanisms of SGLT2 inhibitors.

## 5 Conclusion

This study systematically explored the potential mechanisms and clinical effects of SGLT2 inhibitors in HFpEF patients with hyperuricemia using network pharmacology, clinical statistics, and machine learning. A total of 32 core target genes, including GAPDH, CASP3, and ICAM1, were identified, involved in oxidative stress, apoptosis, and inflammatory pathways. Functional enrichment and molecular docking analyses suggest multi-target effects, particularly on MAPK1 and other cardiovascular and metabolic pathways.

Clinically, SGLT2 inhibitor treatment was associated with exploratory improvements in hemoglobin, urea, left ventricular ejection fraction, and serum creatinine, but no statistically significant reduction in serum uric acid was observed. Machine learning models demonstrated exploratory predictive potential, with creatinine and uric acid as key predictive variables. These findings provide a preliminary mechanistic framework for SGLT2 inhibitors in HFpEF-HUA comorbidity and generate hypotheses for future research. Larger, prospective, multicenter studies with long-term follow-up and experimental validation are warranted to clarify the efficacy and mechanisms of SGLT2 inhibitors in this patient population.

## Data Availability

The datasets used in this study contain sensitive patient information and are therefore not publicly available. Access is restricted in accordance with institutional and ethical regulations to protect patient confidentiality. Requests to access these datasets will be considered only under strict approval by the corresponding author and the institutional ethics committee. Interested researchers may contact the corresponding author at: 2907509542@qq.com.
