# Supplementary material for: The impact of SGLT2 inhibitors on uric acid levels in patients with heart failure with preserved ejection fraction: integration of mechanisms and clinical evidence
Source: Front Cardiovasc Med. 2026 Aug 7;13:1726611. doi: 10.3389/fcvm.2026.1726611 (PMC13494683; doi:10.3389/fcvm.2026.1726611)
Supplement: Supplementary file 1 [file Image1.pdf]

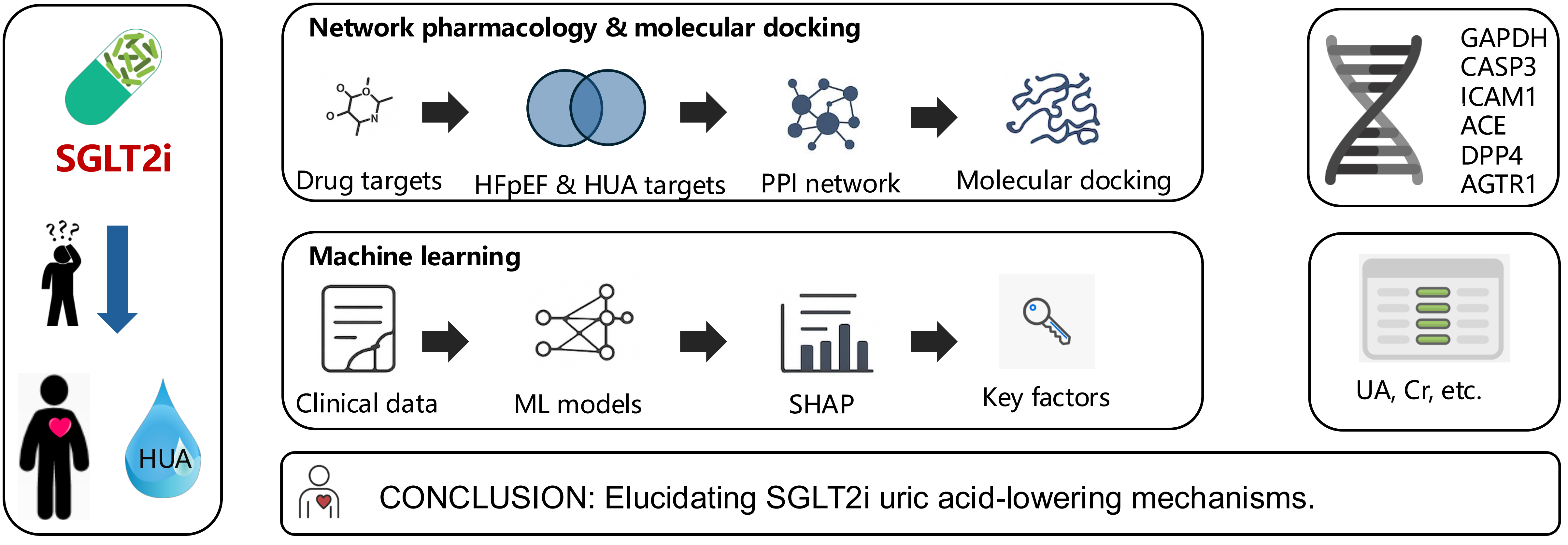
SGLT2i
HUA
Network pharmacology & molecular docking
Drug targets
HFpEF & HUA targets
PPI network
Molecular docking
GAPDH
CASP3
ICAM1
ACE
DPP4
AGTR1
Machine learning
Clinical data
ML models
SHAP
Key factors
UA, Cr, etc.
CONCLUSION: Elucidating SGLT2i uric acid-lowering mechanisms.
